# Individualized Breast Surveillance in a Young Hodgkin Lymphoma Survivor With High-Risk Features

**DOI:** 10.7759/cureus.96776

**Published:** 2025-11-13

**Authors:** Tiffany Kanter

**Affiliations:** 1 Hematology, Oncology and Stem Cell Transplantation, Columbia University, New York, USA

**Keywords:** adolescent and young adult, brca1, breast mass, chest radiation, hereditary breast and ovarian cancer, high-risk features, hodgkin lymphoma, individualized surveillance, risk-based follow-up, survivorship care

## Abstract

Survivors of pediatric Hodgkin lymphoma have an elevated lifetime risk of secondary breast malignancy, particularly those who received chest-directed radiation before age 30. This report describes a 19-year-old female patient, three years post-therapy for stage IIB Hodgkin lymphoma treated with 30 Gy mediastinal radiation, who presented within a structured survivorship program for evaluation of a self-detected, firm, mobile, non-tender left breast mass. Physical examination confirmed a 2 cm lesion in the upper outer quadrant without overlying skin changes, nipple inversion, or lymphadenopathy. Given her therapeutic radiation exposure and significant family history of early-onset breast and ovarian cancers, targeted breast ultrasound and bilateral magnetic resonance imaging (MRI) with contrast were performed for characterization.

Imaging findings were consistent with a benign fibroadenoma, and short-interval follow-up was arranged in accordance with American College of Radiology (ACR) and National Comprehensive Cancer Network (NCCN) guidance for individualized surveillance of high-risk patients. The survivorship evaluation also identified an unaddressed need for germline testing, and subsequent genetic analysis confirmed a pathogenic BRCA1 mutation.

This case illustrates the importance of risk-stratified, multidisciplinary assessment of new breast findings in adolescent and young adult (AYA) cancer survivors. Individualized, radiation-sparing imaging strategies coupled with comprehensive survivorship follow-up enable early detection, genetic risk identification, and longitudinal care coordination for patients at dual risk from prior therapy and hereditary predisposition.

## Introduction

Survivors of pediatric Hodgkin lymphoma represent a growing population with distinct long-term health risks. These include cardiopulmonary toxicity, endocrine dysfunction such as radiation-induced hypothyroidism, and secondary malignancies involving the breast, thyroid, or lung [1,2]. Advances in therapy have markedly improved survival, yet chest-directed radiation remains a leading contributor to secondary breast cancer development. Among females who received chest radiation before age 30, the cumulative incidence of breast cancer approaches 20% by mid-adulthood, a risk comparable to that of BRCA1 mutation carriers [1].

Both the American College of Radiology (ACR) and the National Comprehensive Cancer Network (NCCN) recommend early and enhanced screening protocols for these high-risk survivors, including breast MRI beginning at age 25 or eight years post-treatment, whichever occurs later [3,4]. These guidelines emphasize a multidisciplinary approach integrating oncology, radiology, genetics, and survivorship care to identify late effects and optimize long-term outcomes.

This report presents a case from a structured survivorship program illustrating how prior therapeutic radiation and hereditary cancer predisposition guided the diagnostic evaluation of a new breast mass in a young Hodgkin lymphoma survivor. The case underscores the importance of systematic, guideline-based assessment to differentiate benign lesions from potential secondary malignancies in this high-risk group. Although the lesion in this report was benign, both prior mediastinal radiation and the later-confirmed BRCA1 mutation independently elevate lifetime breast-cancer risk. Chest-irradiated Hodgkin survivors can reach incidences comparable to hereditary-carrier populations, suggesting overlapping rather than distinct risk pathways [1, 5].

## Case presentation

A 19-year-old female patient presented for evaluation of a newly discovered lump in her left breast during an unscheduled visit within a structured cancer survivorship program. She had noticed the mass during self-examination two weeks earlier. The lump was firm, mobile, and non-tender. She denied pain, nipple discharge, overlying skin changes, fever, weight loss, or other systemic symptoms.

Medical history

At age 14, the patient had been diagnosed with stage IIB nodular sclerosis classical Hodgkin lymphoma involving the mediastinum and was treated with combination chemotherapy (adriamycin, bleomycin, vinblastine, dacarbazine) followed by 30 Gy involved-field chest radiation [1]. She achieved complete remission at age 16 and remained disease-free for three years. She was followed annually within a multidisciplinary survivorship program, receiving recommended evaluations including thyroid function testing, echocardiography, and breast surveillance in accordance with the Children’s Oncology Group Long-Term Follow-Up Guidelines for Survivors of Childhood, Adolescent, and Young Adult Cancers [2]. Her only chronic medication was levothyroxine for post-radiation hypothyroidism.

Family history

The patient reported that several maternal relatives had developed breast and ovarian cancers at relatively young ages, suggesting a possible hereditary breast and ovarian cancer syndrome (HBOC) [5]. Neither she nor her family members had previously undergone formal genetic testing. During this visit, the patient’s first encounter with the reporting clinician, the survivorship practitioner identified the absence of germline testing as a potential gap in care and recommended referral for genetic counseling and BRCA testing [5].

Physical examination

On examination, a firm, mobile 2 cm nodule was palpated in the upper outer quadrant of the left breast. There were no overlying skin changes, nipple inversion, tenderness, or fixation to the chest wall. No supraclavicular, cervical, or axillary lymphadenopathy was noted, and the remainder of the examination was unremarkable.

Imaging and diagnostic evaluation

**Figure 1:**
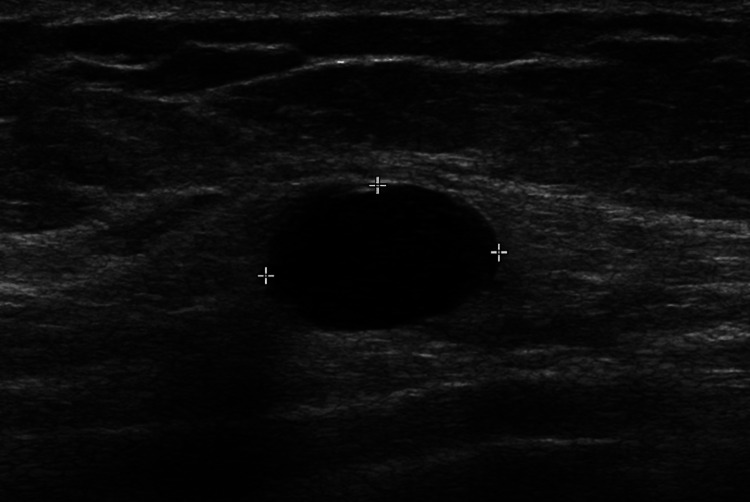
Targeted left breast ultrasound Grayscale ultrasound of the left breast demonstrates a 2 cm oval, well-circumscribed hypoechoic lesion with parallel orientation and smooth margins, consistent with a benign BI-RADS 3 finding in a young post-radiation survivor.

Consistent with the American College of Radiology (ACR) Appropriateness Criteria, targeted breast ultrasound was obtained as the first-line imaging study for a woman under 30 years old [3]. The ultrasound demonstrated a well-circumscribed, oval, hypoechoic lesion with parallel orientation and no posterior acoustic shadowing, features suggestive of a benign fibroadenoma, classified as Breast Imaging Reporting and Data System (BI-RADS) category 3 (Figure 1). 

**Figure 2:**
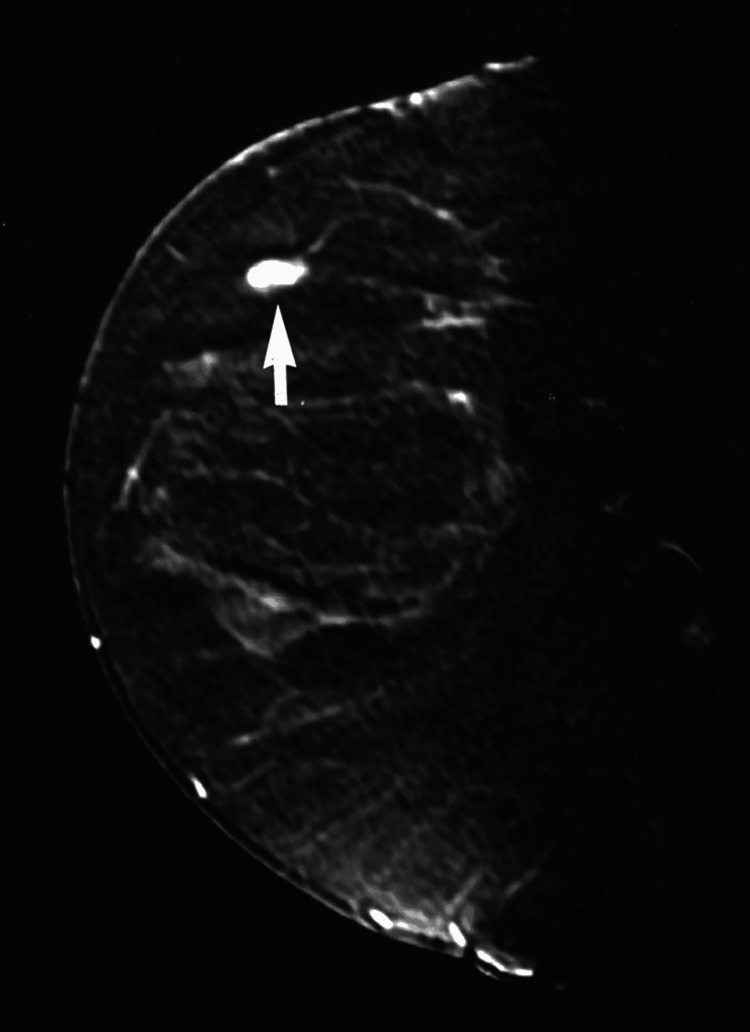
Sagittal T1-weighted post-contrast MRI of the left breast Sagittal MRI of the left breast in a 19-year-old female shows a small, well-circumscribed, oval lesion within the upper outer quadrant (arrow) measuring approximately 2 cm. The lesion demonstrates homogeneous signal intensity without spiculation, abnormal enhancement, or axillary adenopathy, consistent with a benign BI-RADS 3 finding in a post-radiation survivor.

Because of her prior chest irradiation and strong family history of early-onset cancers, bilateral breast MRI with contrast was also performed (Figure 2) [4]. The MRI confirmed a solitary, non-spiculated lesion without abnormal enhancement or axillary adenopathy. Although BI-RADS 3 lesions are typically re-evaluated at six months, a shorter three-month interval was selected in this case to provide closer surveillance consistent with ACR and NCCN recommendations for individualized follow-up in high-risk patients [3,4]. A positron-emission tomography (PET) scan was not indicated, as current NCCN and ACR guidance reserves PET for staging or restaging of known malignancy rather than for initial evaluation of benign-appearing breast findings [3, 4].

Because the lesion was BI-RADS 3 with benign features and remained stable on short-interval imaging, biopsy was deferred; core-needle biopsy would be indicated for interval growth, imaging-clinical discordance, or BI-RADS 4 or higher assessment [6].

Laboratory studies, including complete blood count, thyroid panel, and metabolic profile, were within normal limits (Table 1).

**Table 1:** Baseline laboratory evaluation at presentation Laboratory results demonstrate normal hematologic, thyroid, and metabolic parameters in a 19-year-old female survivor of Hodgkin lymphoma prior to diagnostic imaging. Pregnancy screening was performed routinely before contrast administration and was negative.

Laboratory Test	Result	Reference Range	Clinical Relevance
Complete blood count (CBC)			Confirms normal hematologic recovery and absence of cytopenia following prior therapy
White blood cell count (WBC)	6.4 × 10⁹/L	4.0–10.0 × 10⁹/L	Normal
Hemoglobin (Hgb)	13.8 g/dL	12.0–16.0 g/dL	Normal
Hematocrit (Hct)	40.5 %	36–46 %	Normal
Platelet count (Plt)	286 × 10⁹/L	150–450 × 10⁹/L	Normal
Thyroid panel			Reflects stable thyroid function on levothyroxine replacement following prior radiation exposure
Thyroid-stimulating hormone (TSH)	2.1 mIU/L	0.4–4.0 mIU/L	Normal
Free thyroxine (Free T₄)	1.2 ng/dL	0.8–1.8 ng/dL	Normal
Basic metabolic profile (BMP)			Demonstrates normal renal and metabolic status prior to MRI with contrast administration
Sodium (Na)	139 mmol/L	135–145 mmol/L	Normal
Potassium (K)	4.2 mmol/L	3.5–5.0 mmol/L	Normal
Chloride (Cl)	103 mmol/L	98–107 mmol/L	Normal
Carbon dioxide (CO₂)	25 mmol/L	22–29 mmol/L	Normal
Blood urea nitrogen (BUN)	10 mg/dL	7–20 mg/dL	Normal
Creatinine (Cr)	0.82 mg/dL	0.5–1.1 mg/dL	Normal
Glucose (fasting)	88 mg/dL	70–99 mg/dL	Normal
β-human chorionic gonadotropin (β-hCG, quantitative)	< 1 mIU/mL	< 5 mIU/mL (non-pregnant female)	Confirms non-pregnant status before contrast MRI

Follow-up and outcomes

At three-month re-evaluation, repeat ultrasound and MRI showed no interval change in lesion size or morphology. The patient remained asymptomatic and resumed her regular annual survivorship follow-up, with plans for continued alternating ultrasound and MRI surveillance consistent with ACR and NCCN guidance for high-risk patients [3,4]. As part of the care plan established during that visit, she completed germline genetic testing, which revealed a pathogenic BRCA1 mutation. She subsequently participated in genetic counseling to discuss ongoing screening and risk-reduction strategies tailored to her dual risk from prior radiation exposure and hereditary predisposition.

Patient consent

Written informed consent was obtained from the patient for publication of this case report and accompanying clinical details.

## Discussion

This case demonstrates how comprehensive survivorship care can identify critical gaps in genetic risk assessment and guide tailored diagnostic strategies for breast abnormalities in young Hodgkin lymphoma survivors.

Radiation-associated breast cancer risk

Among female survivors who received ≥20 Gy of chest radiation before age 30, the standardized incidence ratio for breast cancer is 13- to 55-fold higher than in the general population [1]. Risk is amplified by younger age at exposure, higher dose, and concomitant anthracycline therapy. In this case, structured survivorship follow-up identified a missed opportunity for genetic testing and directly shaped how the breast finding was evaluated. Both prior radiation and the BRCA1 mutation likely contributed to elevated cumulative risk; these factors are known to exert overlapping, rather than independent, effects [1, 5].

Diagnostic rationale

Ultrasound is the preferred first-line imaging modality for women under 30 because it avoids additional radiation and accurately distinguishes cystic from solid lesions [3]. Magnetic resonance imaging (MRI) complements ultrasound in high-risk survivors by improving sensitivity for small or multifocal lesions, especially in dense breast tissue [4]. The decision to shorten the follow-up interval from six to three months was supported by ACR and NCCN recommendations, allowing individualized surveillance for high-risk patients. The stability of the lesion over this interval justified continued non-invasive management. A PET scan was not warranted, as current guidelines limit its use to staging of confirmed malignancy rather than for initial evaluation of benign-appearing breast findings [3, 4].

Genetic evaluation and survivorship practitioner role

During the survivorship encounter, the practitioner identified the lack of prior genetic assessment as a deviation from best practice for high-risk survivors. The patient had been followed annually in survivorship care; however, this visit represented her first encounter with the reporting clinician, who recognized the gap and initiated referral in accordance with NCCN recommendations [5]. Germline testing confirmed a BRCA1 mutation, validating the need for enhanced surveillance. This outcome underscores the role of survivorship practitioners in recognizing hereditary risk, coordinating multidisciplinary evaluation, and ensuring adherence to evolving survivorship guidelines.

Psychosocial and educational considerations

Adolescent and young adult (AYA) cancer survivors frequently transition between pediatric and adult care settings, a period during which risk-based follow-up can lapse. Survivorship programs play a critical role in bridging this gap by offering continuity, anticipatory guidance, and proactive screening for late effects. This case underscores that beyond physical surveillance, survivorship encounters provide opportunities for risk communication, psychosocial support, and education that empower patients to participate actively in their long-term health. Incorporating genetic risk review into routine survivorship assessments reinforces shared decision-making and ensures timely evaluation consistent with the Children’s Oncology Group Long-Term Follow-Up Guidelines, which emphasize lifelong risk-based care coordination and transition planning for adolescent and young adult survivors [2].

## Conclusions

Breast masses in young survivors of Hodgkin lymphoma require prompt, structured evaluation because prior chest radiation significantly increases the risk of secondary malignancy. In this case, adherence to ACR and NCCN guidelines enabled safe, non-invasive management while ensuring high-risk surveillance. The identification of a BRCA1 mutation through advanced survivorship assessment highlights the importance of comprehensive survivorship care that integrates genetic evaluation, multidisciplinary coordination, and individualized follow-up.
